# Systemic allergic contact dermatitis to nickel presenting predominantly as severe hand dermatitis, successfully controlled with upadacitinib: A case report

**DOI:** 10.1177/2050313X261446061

**Published:** 2026-04-26

**Authors:** Parisa Mirzajani, Joel G. DeKoven, Anastasia Shamsuyarova

**Affiliations:** 1 Thunder Bay Regional Health Science Centre, Northern Ontario School of Medicine, ON, Canada; 2 Sunnybrook Health Sciences Centre, University of Toronto, ON, Canada

**Keywords:** nickel, systemic allergic contact dermatitis, Janus kinase inhibitor, JAK inhibitors, case report

## Abstract

Systemic allergic contact dermatitis to nickel is a challenging condition often presenting as severe hand dermatitis. This case report details the clinical course of a 33-year-old male truck driver with severe hand dermatitis secondary to systemic allergic contact dermatitis to nickel. Despite significant improvement with avoidance, the patient experienced recurrent flares due to ongoing accidental nickel exposure. Upadacitinib provided an effective salvage strategy. The case underlines challenges of managing systemic allergic contact dermatitis.

## Introduction

Systemic allergic contact dermatitis (ACD) is a type IV hypersensitivity reaction driven by sensitized T-cells, resulting from systemic absorption of allergens like nickel – a prevalent metal which is found in tools, jewelry, and certain food.^1,2^ Unlike localized ACD, systemic ACD arises when nickel enters the body through ingestion, inhalation, or transcutaneous routes, triggering a widespread or localized inflammatory response that can exacerbate pre-existing dermatitis or manifest as new lesions, often in areas like the hands.^3,4^ Nickel is one of the most common allergens implicated in ACD, with prevalence rates of nickel sensitization estimated at 17.5% in North America^5^ and slightly lower in Europe due to EU Nickel Directive.^6^ Systemic ACD to nickel can be life-altering, resulting in job and income loss.^7^

In nickel-induced ACD, activation of the Janus kinase (JAK)/STAT signaling pathway appears to play a key pathogenic role.^8^ Given the challenges many patients face in maintaining strict allergen avoidance, upadacitinib, a selective JAK 1 inhibitor, has emerged as a potential salvage therapy to control systemic flares when inadvertent exposures occur.^9^

This case report centers on a 33-year-old male truck driver with a long-standing history of severe hand dermatitis due to systemic uncontrolled ACD to nickel.

## Case presentation

The patient is a 33-year-old male truck driver with a history of hand dermatitis since age 15.

**Figure 1.**
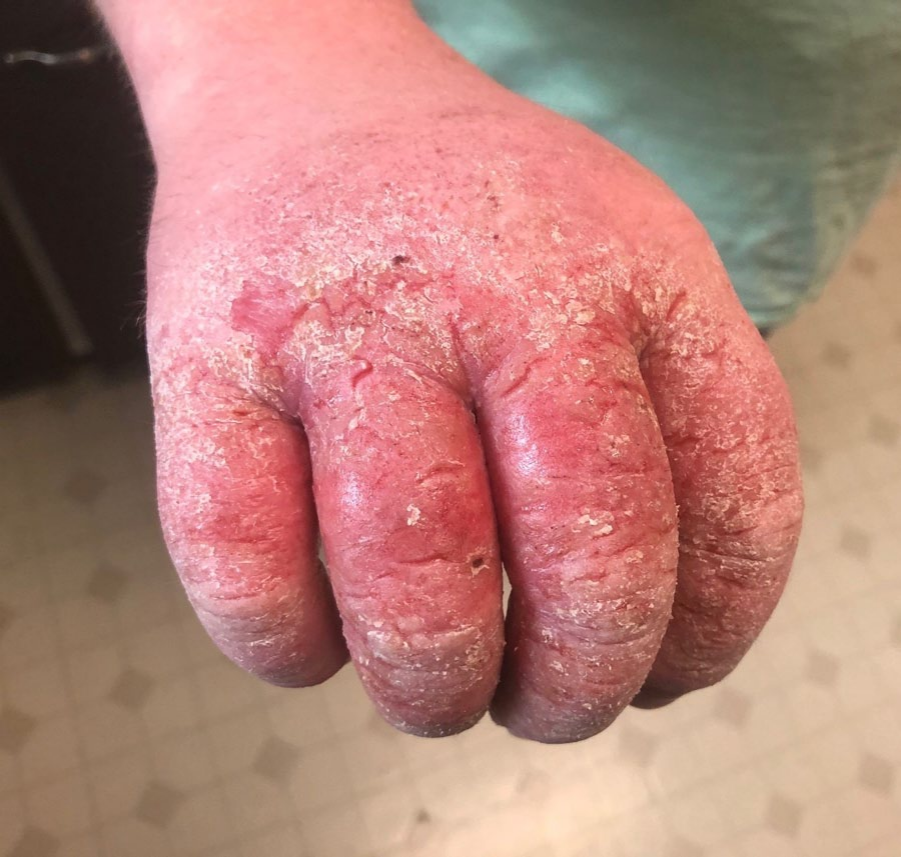
Patient’s hand during an active flare-up.

The patient has worked as a truck driver for a roofing company. He denied a personal or family history of atopy or other skin conditions. His dermatitis has progressively worsened since 2015, with severity rated as 10/10 at times, characterized by marked swelling, splits in the palms, and fingers (Figure 1). He presented multiple times to the Emergency Department at a local hospital and was frequently provided with oral antibiotics and high-dose courses of prednisone. His first dermatology assessment took place on July 8, 2020.

The initial impression was severe hand eczema with a possible ACD component. Due to work schedule and difficult social situation, the patient could not commit to patch testing at first. Management with multiple topical corticosteroids, calcineurin inhibitors and PDE4 inhibitors as well as systemic alitretinoin and methotrexate, yielded minimal improvement. He experienced repeated severe flares, requiring high-dose systemic corticosteroids.

Subsequently, he was referred to the Occupational Health Department at an academic center in 2021. The patient was patch tested to the North American Contact Dermatitis Group Screening and Supplement series (Chemotechnique Diagnostics, Vellinge, Sweden); pieces of his gloves, tested wet and occluded for 7 days; his moisturizer; and his topical therapies. Readings were performed at 48 and 168 h. At the final reading, reactions were noted to:

- *Carba Mix 3% in petrolatum (pet) 2+ and Thiuram Mix 1% pet 2+*: Rubber accelerators found in gloves and work tools.
- *Nickel sulfate hexahydrate 2.5% and 5% pet, 2*+: Associated with metal belt buckles and work-related tools, and possibly indicating systemic nickel contact dermatitis.
- *Diazolidinyl urea 1% pet 1+ and imidazolidinyl urea 2% pet 1*+: Formaldehyde-releasing preservatives in personal care products.
- *Phenoxyethanol 1% pet 1*+: A preservative in hygiene/personal care products (clinical significance uncertain).

Strict nickel avoidance was emphasized, with recommendations for protective non-rubber gloves, avoiding direct skin contact with nickel-containing items, and following a low-nickel diet aided by the Nickel Navigator app (Nicek Navigator developed by Rebelytic R&D, Inc.). Prior to the identification of his allergy, he consumed canned foods such as Campbell’s soup almost daily, further raising the possibility of systemic ACD.

The patient experienced almost complete clearance of his skin with a strict low-nickel diet. However, due to difficulty maintaining strict dietary adherence, he continued to have intermittent flares. Once, he presented urgently with a flare following the consumption of two cans of beer at a social gathering, having run out of his usual bottled beer. He reported similar relapses when using a Yeti mug (has 8% nickel) after dining at friends’ homes. Overall, while dietary avoidance reduced both frequency and severity of symptoms, complete adherence was challenging, and periodic flares persisted with lapses in avoidance.

In early 2024, after continued flares, upadacitinib 15 mg a day was initiated. He reported remarkable improvement, with clear hands and no new flares.

**Figure 2.**
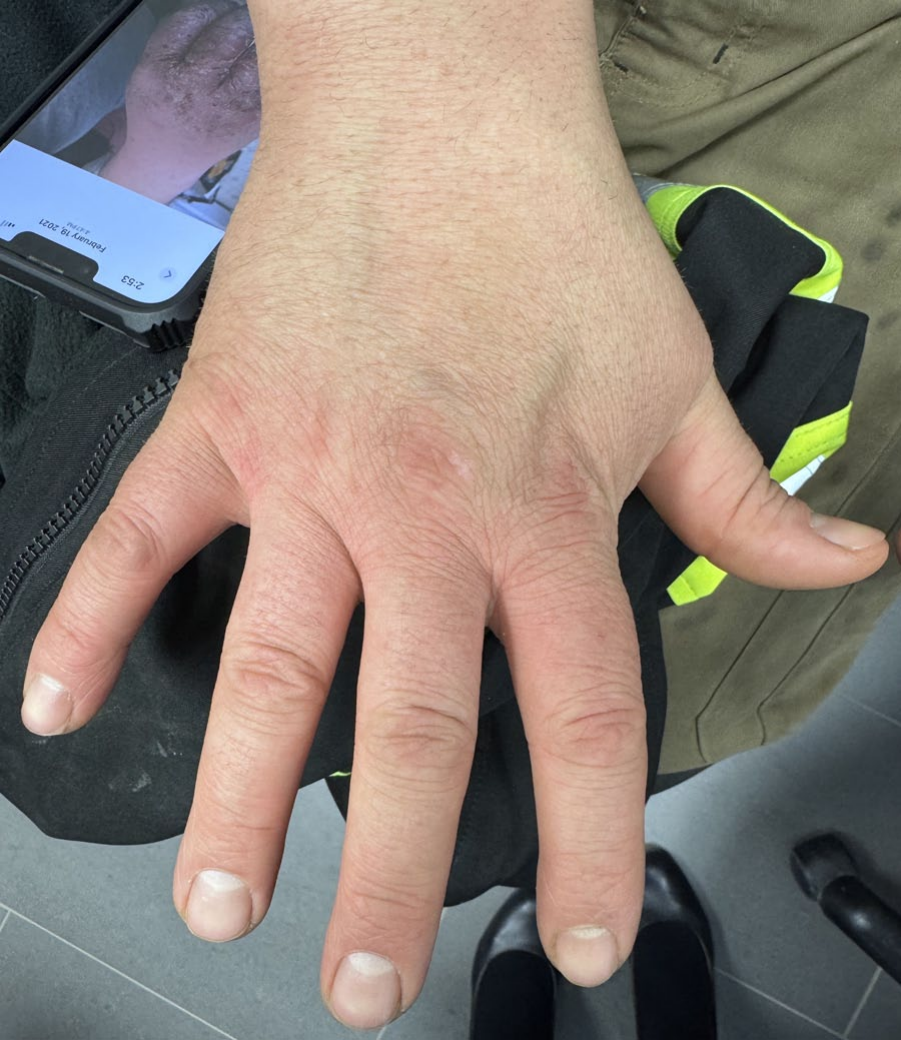
Patient’s hands in remission while on upadacitinib.

The patient remains well-controlled on upadacitinib, with continued allergen avoidance and occupational modifications (Figure 2).

## Discussion

This case underlines the intricate challenges associated with managing systemic ACD to nickel, exacerbated by both occupational and dietary exposures.

Upadacitinib, a JAK inhibitor, achieved significant clinical improvement, with the clearing of the patient’s hands and concomitant enhancement of quality of life. This outcome mirrors findings in other ACD cases where biologic or targeted therapies have proven helpful, particularly when allergen avoidance is suboptimal.^9–11^

Our case highlights the need for continued vigilance regarding nickel exposure. Uncontrolled ACD can severely impair a patient’s ability to work and earn income. This case also underscores the importance of integrating patch testing, personalized avoidance strategies, equitable access to advanced therapies and public health measures to diminish nickel sensitization in North America.

## Conclusion

Systemic ACD to nickel, presenting as severe hand dermatitis, is a debilitating condition requiring a multifaceted approach, including allergen identification, avoidance, and tailored therapy. This case underscores the challenges of managing such patients, particularly in the context of occupational exposure and financial constraints. Patch testing is essential for identifying allergens, while advanced therapies like upadacitinib offer hope for refractory cases.
